# Accuracy of Nurse-Performed Lung Ultrasound in Patients With Acute Dyspnea: A Prospective Observational Study

**DOI:** 10.1097/MD.0000000000002925

**Published:** 2016-03-07

**Authors:** Nicola Mumoli, Josè Vitale, Matteo Giorgi-Pierfranceschi, Alessandra Cresci, Marco Cei, Valentina Basile, Barbara Brondi, Elisa Russo, Lucia Giuntini, Lorenzo Masi, Massimo Cocciolo, Francesco Dentali

**Affiliations:** From the Department of Internal Medicine, Ospedale Civile di Livorno, Livorno (NM, AC, MC, VB, BB, ER, LG, LM, MC), Department of Internal Medicine, Ospedale di Circolo, Varese (JV, FD), and Emergency Department, Ospedale della Val d’Arda, Piacenza (MGP), Italy.

## Abstract

In clinical practice lung ultrasound (LUS) is becoming an easy and reliable noninvasive tool for the evaluation of dyspnea. The aim of this study was to assess the accuracy of nurse-performed LUS, in particular, in the diagnosis of acute cardiogenic pulmonary congestion.

We prospectively evaluated all the consecutive patients admitted for dyspnea in our Medicine Department between April and July 2014. At admission, serum brain natriuretic peptide (BNP) levels and LUS was performed by trained nurses blinded to clinical and laboratory data. The accuracy of nurse-performed LUS alone and combined with BNP for the diagnosis of acute cardiogenic dyspnea was calculated.

Two hundred twenty-six patients (41.6% men, mean age 78.7 ± 12.7 years) were included in the study. Nurse-performed LUS alone had a sensitivity of 95.3% (95% CI: 92.6–98.1%), a specificity of 88.2% (95% CI: 84.0–92.4%), a positive predictive value of 87.9% (95% CI: 83.7–92.2%) and a negative predictive value of 95.5% (95% CI: 92.7–98.2%). The combination of nurse-performed LUS with BNP level (cut-off 400 pg/mL) resulted in a higher sensitivity (98.9%, 95% CI: 97.4–100%), negative predictive value (98.8%, 95% CI: 97.2–100%), and corresponding negative likelihood ratio (0.01, 95% CI: 0.0, 0.07).

Nurse-performed LUS had a good accuracy in the diagnosis of acute cardiogenic dyspnea. Use of this technique in combination with BNP seems to be useful in ruling out cardiogenic dyspnea. Other studies are warranted to confirm our preliminary findings and to establish the role of this tool in other settings.

## Abstract 2

Supplemental Digital Content is available in the text

## INTRODUCTION

Dyspnea, one of the most common symptoms in clinical practice, affects 25% of patients in ambulatory care and more than 50% of patients admitted to acute tertiary hospitals.^1,2^ Prompt identification of the correct cause of dyspnea may optimize the management and improve the outcome of these patients.

Diffuse use of ultrasonography as an “ultrasound stethoscope” is rapidly becoming a reality into medical education.^3^ In particular lung ultrasound (LUS), once considered unconceivable, is emerging as a bedside imaging tool for evaluating the whole spectrum of thoracic diseases: pulmonary congestion and fibrosis, pneumonia, pneumothorax, pleural effusion, tumors, and pulmonary embolism. Growing evidence showed that LUS is more accurate than physical examination and conventional chest radiography for rapid detection of pulmonary congestion due to heart failure.^4–7^ Also in nonphysicians hands chest ultrasound could discriminate between cardiac and noncardiac dyspnea.^5^ Today, trained nurses are already able to perform ultrasound in the placement of central,^8^ peripheral line,^9^ and for the focused assessment of urologic,^10^ obstetric,^11^ and cardiocirculatory^12,13^ diseases. On the other hand, there is only a single pilot study that evaluates the utility of nursing LUS in the broad differential diagnosis of dyspnea.^14^ Thus, we performed a prospective study to assess the accuracy and of nurse-performed LUS in the diagnosis of acute cardiogenic dyspnea.

## MATERIALS AND METHODS

The study was conducted and reported according to the Standards for Reporting of Diagnostic Accuracy Studies initiative, which established reporting guidelines for diagnostic accuracy studies to improve the quality of reporting.^15^ Local ethics committee approved the study protocol, and a written consent was obtained from all the study participants.

### Aim of the Study

The aim of the study was to evaluate the accuracy of the nurse-performed LUS in the diagnosis of cardiogenic dyspnea. Subsequently as a secondary aim of the study the accuracy of a combination of LUS and brain natriuretic peptide (BNP) was assessed.

### Sonographers Training

Five nurses of the Internal Medicine Department of Livorno Hospital, Italy, were trained in lung ultrasonography in order to identify typical signs of interstitial syndrome of cardiogenic pulmonary congestion. The 4-week training course consisted of a 8 hours of didactic lectures followed by overall 20 hours of practice using living models and 4 hours of chest ultrasound image review.

### Study Population

All the consecutive patients admitted to the Emergency Department of Livorno Hospital, Italy, for dyspnea between April and July 2014 were prospectively evaluated. Dyspnea was considered as a conscious shortness of breath with a respiratory rate more than 24 breaths per minute, an oxygen saturation of less than 92% or been started on oxygen therapy. Only patients in whom a hospital admission was planned were considered eligible for the study purpose. Conversely, patients were ineligible if they were aged <18 years, had trauma or known pneumothorax, required dialysis or intensive observation, had severe instability of vital signs, refused consent or had technical limitations for the ultrasound examination (mental disability or extreme agitation) (see Supplementary Appendix for details).

### Study Procedures

All eligible patients underwent, within 90 minutes from admission, a bedside LUS performed by a trained nurse. Results were registered, sealed, and stored. Nurses were unaware of primary clinical assessment, diagnostic tests (laboratory and radiological) and treatments performed in the Emergency and Medicine Department. To not break the blind protocol, patients were asked to not provide information on their medical history to the operators during LUS.

### Lung Ultrasound Diagnosis: LUS

**FIGURE 1:**
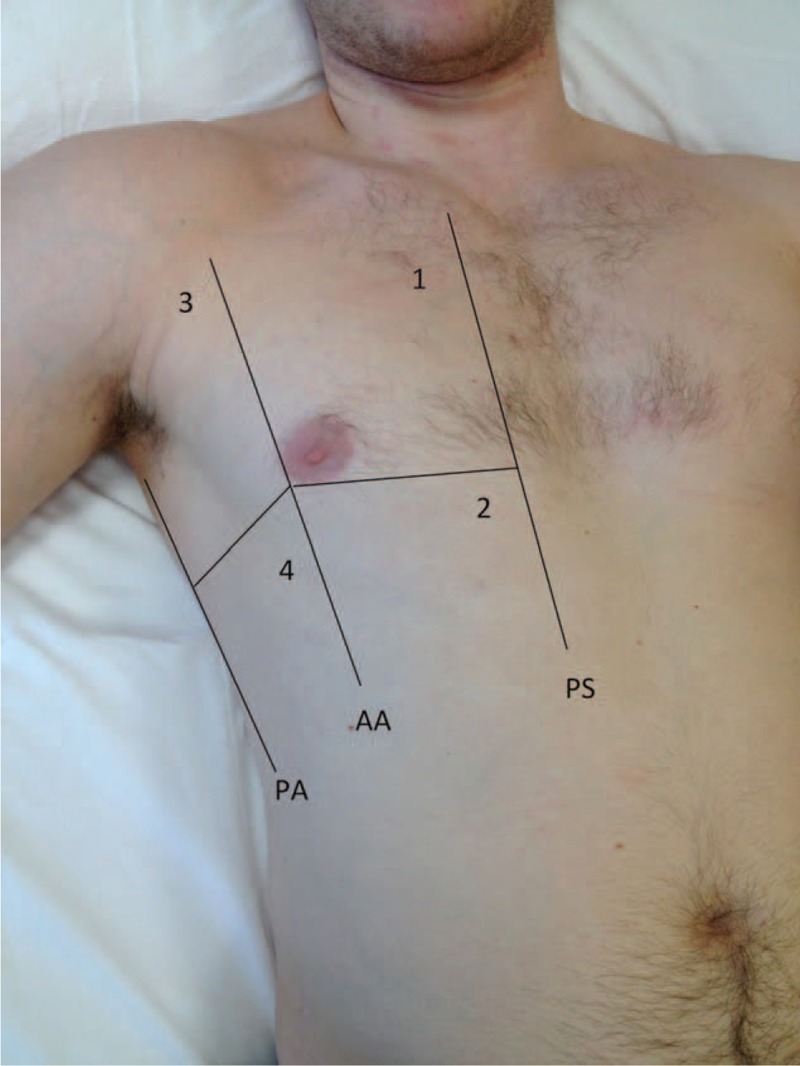
The basic eight-region of the chest for sonographic technique.

All bedside LUS were performed using a GE Vivid S5 (GE Healthcare, Milwaukee, WI) with a 2- to 5.5-MHz curved-array transducer (M4C-RS). According to the International evidence-based recommendations for point-of-care LUS,^16^ after individuating pleural line, operators searched the artifacts. Normal lung artifacts, so called A-line or A-pattern, are the repetition of the pleural line appearing as horizontal hyperechoic lines parallel to the pleural line, due to air block in ultrasound diffusion among pulmonary tissue. The B-lines, or comet-tail signs, are defined as laser-like, vertical hyperechoic reverberation artifacts that arise from the pleural line, extend to the bottom of the screen without fading, and move synchronously with lung sliding. These lines are related to the presence of extravascular fluid in the lung and the rate was considered directly proportional. The term “B-pattern” should be used to describe interstitial pulmonary syndrome through the presence of multiple diffuse bilateral B-lines. A positive region is defined by the presence of three or more B-lines in a longitudinal plane between two ribs. The consensus process defined the basic eight-region of the chest for sonographic technique (Figure 1): 4 anterior and 4 lateral. LUS was positive for cardiogenic pulmonary congestion if B-patterns were observed in 2 or more areas bilaterally.^16^ Similar B-patterns are observed in many acute and chronic conditions with diffuse interstitial involvement. However, some sonographic signs other than B-lines (eg, alterations of the pleura, as small subpleural consolidations or evident thickening; “spared areas,” defined as areas of normal sonographic lung appearance surrounded by areas of multiple B-lines; and large consolidations of various size) are useful to differentiate the B-pattern of cardiogenic pulmonary edema from acute respiratory distress syndrome (ARDS) or pulmonary fibrosis.^17^

### Brain Natriuretic Peptide (BNP)

BNP levels were measured at the time of the enrollment in all patients (Architect I2000SR, Abbott Diagnostics, Abbott Park, Illinois, USA). According to the literature,^18^ value <100 pg/mL was within the normal range and ruled out congestive heart failure (CHF), whereas value ≥400 pg/mL strongly suggests heart failure while BNP levels <400 pg/mL are not diagnostic.

### Reference Test: Overall Final Diagnosis

Standard reference test was the overall final diagnosis which indicated the leading cause of patient's dyspnea. The final leading diagnosis of dyspnea was assessed by 2 external independent physicians (JV and FD) through review of the entire medical record of each patients: clinical history and assessments, clinical course and evolution, diagnostic tests (echocardiography, biochemical data, and other imaging studies), treatment outcome using European Society of Cardiology (ESC) guidelines algorithm^19^ (for details see Supplementary Appendix). In case of a disagreement a third physician (MC) was consulted and adjudicated the case. All external physicians were masked to LUS results.

In case of concomitant presence of more than one causes of dyspnea (eg, cardiogenic dyspnea and acute exacerbation of a chronic obstructive pulmonary disease (COPD)), patient was considered to have cardiogenic dyspnea for the study purpose.

### Statistical Analysis

Since there are only sparse data on the accuracy of LUS performed by trained nurses in the diagnosis of acute decompensated CHF a formal calculation of the study sample could not be performed. Thus a convenience sample of at least 200 patients has been chosen.

Continuous variables were summarized as mean and standard deviation, and noncontinuous variables as frequencies and percentages. Diagnostic accuracy, sensitivity, specificity, positive and negative predictive values, and likelihood ratios (LR) of nurse-performed LUS with their 95% CIs were calculated, using the overall final diagnosis as the reference test. The posttest probability of having or not having the target disorder in the case of positive or negative nurse-performed LUS was calculated. Subsequently, the accuracy of LUS in combination with BNP was evaluated. Statistical analyses were performed using SPSS 15.0 software (SPSS, Inc., Chicago, IL).

## RESULTS

**FIGURE 2:**
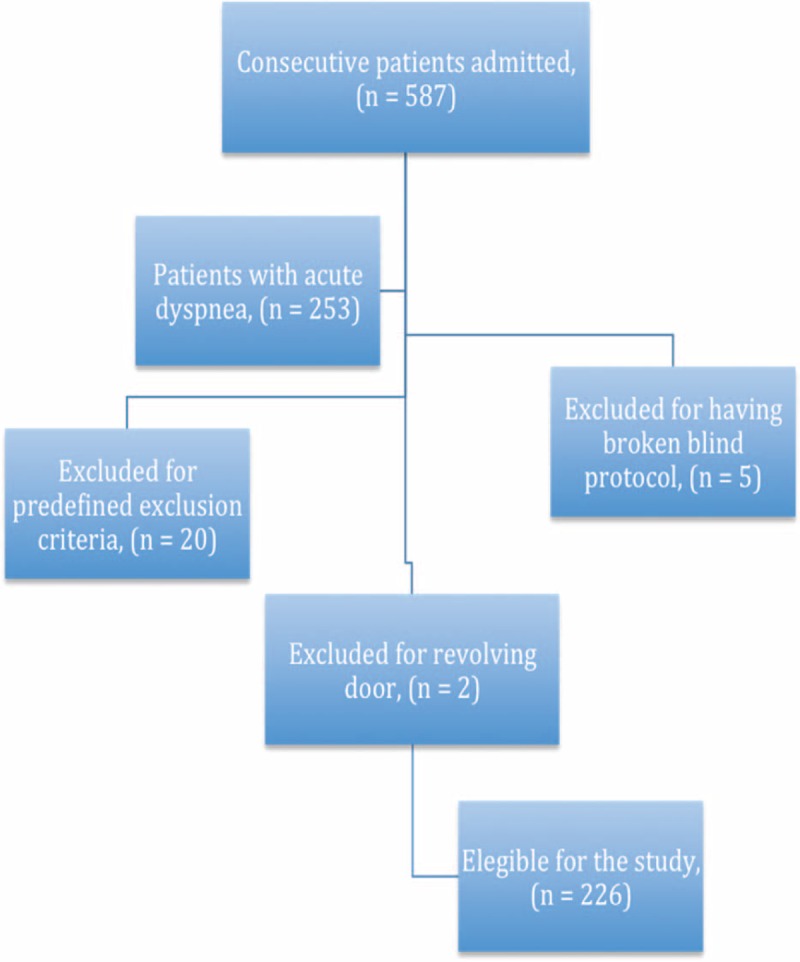
Summary of selection.

From April to July 2014, 253 of the 587 patients admitted to the Internal Medicine Department patients had acute dyspnea (43%). Twenty patients were excluded for one or more predefined exclusion criteria, additional 5 patients were excluded from the analysis from blind protocol violation, having communicated results of prior exams or their medical history, and 2 patients for revolving door in short time (Figure 2). The characteristics of the remaining 226 patients are outlined in Table 1. Ninety-four patients (41.6%) were men and mean age was 78.7 years (±12.7). BNP was not available in 4 patients, was abnormal (≥400 pg/mL) in 119 patients and nondiagnostic in 103 patients. LUS was suspected for cardiogenic dyspnea in 116 patients (51.3%).

**TABLE 1:**
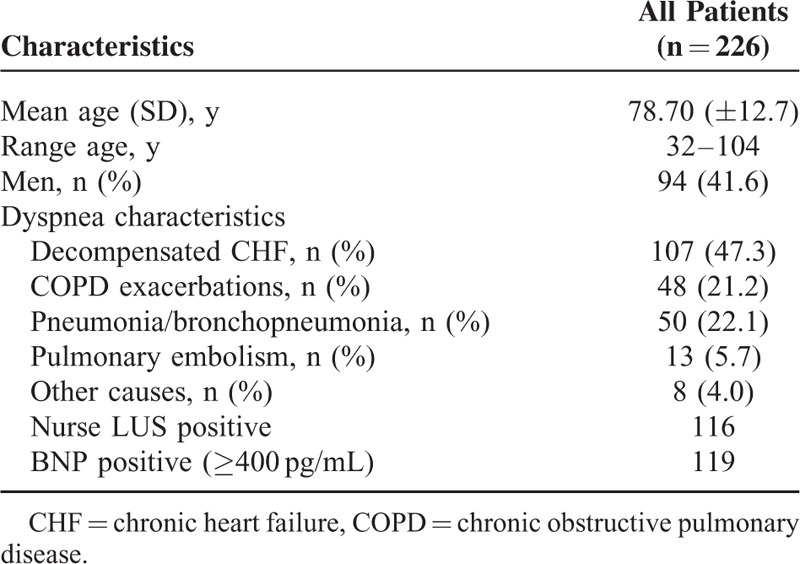
Patient and Dyspnea Characteristics

At the end of the diagnostic work up acute cardiogenic dyspnea was diagnosed in 107 patients (47.3%); 48 (21.2%) had COPD exacerbations, 50 (22.1%) pneumonia, 13 (5.7%) pulmonary embolism and the remaining 8 patients (4%) had other causes as the leading final diagnosis (Table 1). LUS median time execution was 4 minutes, range 2 to 6 minutes.

Nurse-performed LUS, compared to a reference overall diagnosis, showed a sensitivity of 95.3% (95% CI: 92.6–98.1%) and specificity of 88.2% (95% CI: 84.0–92.4%), a positive predictive value of 87.9% (95% CI: 83.7–92.2%) and a negative predictive value of 95.5% (95% CI: 92.7–98.2%). Positive and negative LRs were 8.1 (95% CI: 7.1, 9.1) and 0.05 (95% CI: 0.02, 0.08), respectively. Thus, the posttest probability of having acute cardiogenic dyspnea increased to 88% in case of positive LUS, and it decreased to 4% when the LUS was negative (Table 2). The accuracy of LUS in specific subgroups according to gender and age (<80 and >80 years) is reported in Table 3. In these subgroups LUS had in general a similar accuracy with a perfect sensitivity (100%, 95% CI: 80.8–100%) and negative predictive value (100%, 95% CI: 93.4–100%) in patients younger than 80 years.

**TABLE 2:**
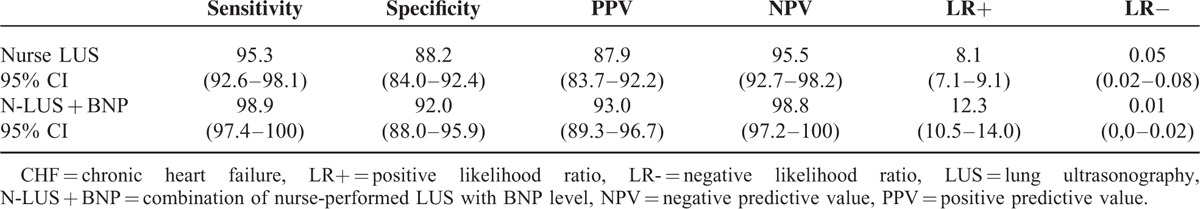
Diagnostic Accuracy of Nurse-Performed LUS for Decompensated CHF

**TABLE 3:**
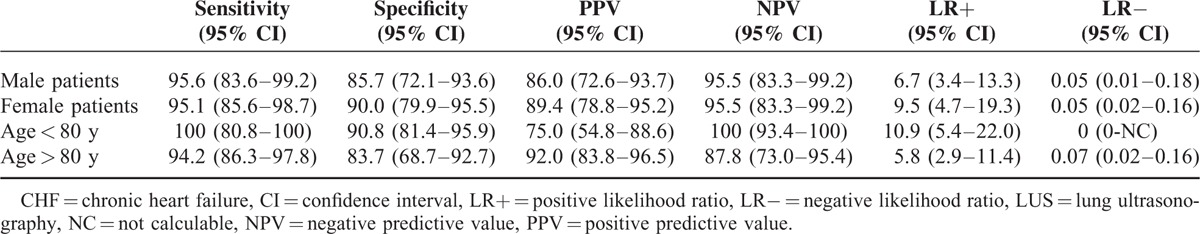
Diagnostic Accuracy of Nurse-Performed LUS for Decompensated CHF in Specific Subgroups of Patients

Subsequently, we tested the accuracy of combining nurse-performed LUS with BNP level using the cut-off 400 pg/mL for BNP and considering as positive any LUS suspected for cardiogenic dyspnea and any BNP ≥400 pg/mL. With these combined test, 51.9% had suspected cardiogenic dyspnea, the sensitivity increased to 98.9% (95% CI: 97.4–100%), the negative predictive value increased to 98.8% (95% CI: 97.2–100%) with a corresponding negative LR of 0.01 (95% CI: 0.0, 0.07) (Table 2).

## DISCUSSION

Cardiogenic dyspnea is one of the most common causes of dyspnea.^1,2^ A substantial proportion of patients presenting to the emergency and medical departments with dyspnea has an incorrect diagnosis and inappropriate treatment of this disease which increases the number of deaths and length of hospital stay.^20^ Many doubts rise about the true value of clinical examination in patients admitted with suspect of cardiogenic dyspnea.^21–23^ On the other hand, especially in the last few years, a number of studies consistently demonstrated a good accuracy of LUS in this field.^4–7,16^ In particular, in patients presenting with dyspnea, the easy and reliable approach of chest ultrasound rapidly allowed to differentiate with good reproducibility between acute cardiogenic and noncardiogenic dyspnea.^4–7,16^

Results of our study suggested a potential role of LUS in this setting even in nonexpert hands. Overall, LUS performed by nurses with a limited clinical and ecographic experience (8 hours of didactic lectures followed by overall 20 hours of practice using living models and 4 hours of chest ultrasound image review) had a good accuracy in the diagnosis of cardiogenic dyspnea. Moreover, this technique, in combination with BNP, seems extremely useful in ruling out the cardiogenic origin of dyspnea with a negative predictive value of 98.8% (95% CI: 97.2–100%) and a negative LR of 0.01 (95% CI: 0.0, 0.07).

Similar results were obtained in a previous study^14^ where the nurse-performed LUS had sensitivity and specificity above 95% for the diagnosis of cardiogenic dyspnea. However, the small study sample (96 patients) and the lack of an established protocol for the diagnosis of dyspnea of cardiac origin limited the validity of their results. Furthermore, in that study, the potential adjunctive role of BNP was not evaluated.

Our results, if confirmed in other larger prospective high quality studies, have potentially significant clinical implications. LUS is an easy to learn and easy to perform, rapid and high accurate technique, in the diagnosis of cardiogenic dyspnea. It could be potentially used in settings where other techniques are not readily available, such as remote locations, at high altitudes, in developing countries with scant resources and capabilities, or even in the out-of-hospital setting (eg, residential care, ambulance, and helicopters) where the availability of a trained physician may be limited. Results of our study suggest that nurse-performed LUS may be a potential useful alternative to the traditional physician-performed LUS. However, nurses should be adequately trained, and at the moment further studies are needed to standardize the training and the setting in which this practice could be safely and efficiently applied.

Our study has some limitations. First, observational studies are at high risk of bias that may affect the internal validity of the results. However several strategies were carried out to minimize the risk: patients were enrolled consecutively and prospectively; the protocol of blindness has been rigidly respected; investigators were properly trained. Second, the study was conducted on a single center and the same evaluation in another setting may give different results, but we tried to reflect real-world practice excluding from the study a few patients only and involving 5 nurses without significant differences in the accuracy among themselves. Third, since only patients in whom a hospital admission was planned were considered eligible for the study purpose a selection bias could not excluded and our results may not be applicable to patients with milder symptoms. Last, our results may be affected by previous treatments. However, we tried to minimize the time between the presentation to the emergency medicine and the examination (<90 minutes) to reduce the likelihood of this potential bias.

In summary, nurse-performed LUS had a good accuracy in diagnosing dyspnea of cardiac origin. Use of this technique in combination with BNP seems to be useful in ruling out cardiogenic dyspnea. Thus, it may be a useful tool to improve diagnostic accuracy and reduce the waiting time between the admission and diagnosis into the overcrowded emergency departments and medical wards. Other studies are warranted to confirm our preliminary findings and to establish the role of this tool in other settings.

## Supplementary Material

Supplemental Digital Content
