# Supplementary material for: Accuracy of Nurse-Performed Lung Ultrasound in Patients With Acute Dyspnea: A Prospective Observational Study
Source: Medicine (Baltimore). 2016 Mar 7;95(9):e2925. doi: 10.1097/MD.0000000000002925 (PMC4782880; doi:10.1097/MD.0000000000002925)
Supplement: Supplemental Digital Content [file medi-95-e2925-s001.doc]

**Appendix**

Inclusion criteria:

- Patients hospitalized with ≥ 18 years of age
- Conscious shortness of breath with a respiratory rate more than 24 breaths per min, an oxygen saturation of less 92% or been started on oxygen therapy
- Consent obtained

Exclusion criteria:

- Patients with < 18 years of age
- Consent refused
- Invasively ventilated
- Traumatic injuries
- Required dialysis or intensive observation
- Severe instability of vital signs
- Technical limitations for LUS: mental disability, extreme agitation, uncooperative

**ESC Guidelines algorithm for Heart failure**

**The**[**ESC**](https://en.wikipedia.org/wiki/European_Society_of_Cardiology)**algorithm weights the following parameters in establishing the diagnosis of heart failure.**

| **Diagnostic assessments supporting the presence of heart failure** |  | | | | |
| --- | --- | --- | --- | --- | --- |
| **Assessment** | | | **Diagnosis of heart failure** |  | |
|  |  |  | **Supports if present** | | **Opposes if normal or absent** |
| **Compatible symptoms** | | | **++** | | ++ |
| **Compatible signs** | | | **++** | | + |
| **Cardiac dysfunction on echocardiography** | | | **+++** | | +++ |
| **Response of symptoms or signs to therapy** | | | **+++** | | ++ |
| **ECG** | |  | | | |
| **Normal** | | |  | | ++ |
| **Abnormal** | | | **++** | | + |
| **Dysrhythmia** | | | **+++** | | + |
| **Laboratory** | |  | | | |
| **Elevated BNP/NT-proBNP** | | | **+++** | | + |
| **Low/normal BNP/NT-proBNP** | | | **+** | | +++ |
| [**Low blood sodium**](https://en.wikipedia.org/wiki/Hyponatremia) | | | **+** | | **+** |
| **Kidney dysfunction** | | | **+** | | + |
| **Mild elevations of troponin** | | | **+** | | + |
| **Chest X-ray** | |  | | | |
| **Pulmonary congestion** | | | **+++** | | + |
| **Reduced exercise capacity** | | | **+++** | | ++ |
| **Abnormal pulmonary function tests** | | | **+** | | + |
| **Abnormal hemodynamics at rest** | | | **+++** | | ++ |
| **+ = some importance; ++ = intermediate importance; +++ = great importance.** | |  | | | |
